# Determination of Paddy Soil Ammonia Nitrogen Using Rapid Detection Kit Coupled with Microplate Reader

**DOI:** 10.3390/toxics10120725

**Published:** 2022-11-25

**Authors:** Xiaoting Liu, Dan Wu, Abbas Ali Abid, Ying Liu, Jianfeng Zhou, Qichun Zhang

**Affiliations:** 1 Key Laboratory of Environment Remediation and Ecological Health, Ministry of Education, College of Environmental & Resource Science, Zhejiang University, Hangzhou 310058, China; 2 College of Biosystems and Food Science, Zhejiang University, Hangzhou 310058, China; 3 Department of Food Nutrition and Detection, Hangzhou Vocational & Technical College, Hangzhou 310018, China; 4 Cixi Environmental Protection Monitoring Station, Ningbo 315300, China

**Keywords:** microplate reader, soil ammonia nitrogen, rapid detection kit, indophenol blue colorimetry

## Abstract

Indophenol blue colorimetry has been widely used for determining soil ammonia nitrogen, but this method has some disadvantages, such as complex reagent preparation, high toxicity, and long colorimetric time. Hence, we aimed to develop a rapid soil ammonia nitrogen determination method using a rapid detection kit. In order to select a suitable extractant, different concentrations of KCl and NaCl solutions were used to extract soil. The ammonia nitrogen content in different types of soils was determined using a rapid detection kit (purchased from Zhejiang Luheng Environmental Technology Limited Company) coupled with a microplate reader. The kit method was compared with the traditional indophenol blue colorimetry method. The results showed no significant difference between the 1 mol·L^−1^ KCl extraction kit method and indophenol blue colorimetry (*p* > 0.05). The linearity of the working curve was smooth, the linear detection range was 0.0–2.00 mg·L^−1^, the average relative standard deviation was 7.00% (n = 5), the standard addition recovery rate was 89.31–118.23%, and the detection limit were was 0.074 mg·L^−1^. We concluded that the 1 mol·L^−1^ KCl extraction kit method can be applied to determine the ammonia nitrogen content of paddy soil with different chemical properties. The 1 mol·L^−1^ KCl extraction kit method has the advantage over indophenol blue colorimetry due to its simple reagent preparation, convenient operation, and shorter detection time (the coloring and colorimetric time for 96 samples was only 30 min using the kit method coupled with a microplate reader). Hence, it has the potential for application due to its rapid determination of soil samples in large quantities.

## 1. Introduction

Organic and inorganic nitrogen are the two main forms of soil nitrogen, and their determination is essential for evaluating soil fertility. Nitrogen fertilizer (mainly urea) is decomposed into ammonia nitrogen by urease after being applied to the soil. A part of the applied ammonia nitrogen can be directly absorbed and utilized by plants [1], while a small amount of ammonia nitrogen is converted into NO_3_^−^ by nitrification and is absorbed by the soil. The rest volatilizes into the atmosphere in the form of NH_3_, which causes a considerable loss of nitrogen from the soil [2]. Previous studies have shown that nitrogen loss caused by ammonia volatilization could reach 9.20–45.30% of applied nitrogen [3,4,5]. When volatile ammonia reaches the atmosphere, it combines with NO_X_ and SO_2_ to form aerosol particles [6,7,8], which are the main components of PM2.5 in the haze. Additionally, when ammonia oxidizes into NO, NO_2_, and N_2_O in the troposphere, NO_X_ returns to the soil and surface water through dry and wet sedimentation, causing soil acidification and water eutrophication [9]. Therefore, it is crucially important to monitor ammonia nitrogen content in the soil.

A number of methods have been developed to determine ammonia nitrogen in water. Some researchers adopted common methods, while the others proposed improved methods for determining ammonia nitrogen in water [10,11]. However, there is no unified national or industrial standard method for determining soil ammonia nitrogen in China. According to the third edition of *Soil Agrochemical Analysis* [12], soil ammonia nitrogen determination methods include the distillation–titration method and indophenol blue colorimetry. The distillation–titration method is suitable for the determination of an ammonia nitrogen concentration of more than 0.2 mg·L^−1^, and the upper detection limit should reach 1000 mg·L^−1^. However, the distillation–titration method has some disadvantages, such as its large reagent consumption, its potential to produce inaccurately high results [13], and its distillation time of 10–20 min, which is not suitable for rapid and large-scale ammonia nitrogen determination. Indophenol blue colorimetry has special issues of sensitivity. Although indophenol blue colorimetry is easier to perform than the distillation–titration method, it requires toxic reagents, such as phenol, and it is time consuming because the chromogenic process requires up to 1 h. In addition, the absorbance is easily disturbed by Cu^2+^, Fe^3+^, Zn^2+^, and other metal ions in soil. Hence, it is necessary to add a masking agent to eliminate the interference of relevant ions. Recently, the fluorescence analysis method [14,15], continuous flow analyzer [16], gas phase molecular absorption spectrometer [17], ammonia electrode [18,19], electrochemical ammonia–nitrogen sensor [20], flow injection analysis [21], ion chromatograph [22], and microplate reader are also being used to determine soil ammonia nitrogen, but the microplate reader has the advantage over conventional instruments due to its enzyme-linked immunosorbent assay. Compared with the traditional ultraviolet spectrophotometer, the operation of the microplate reader is simpler and faster, and it is suitable for analyzing large batches of samples because in colorimetry 96 samples can be analyzed at the same time within 1 min. At present, the microplate reader has been widely used to determine total phosphorus [23], total petroleum hydrocarbons [24], and nitrogen [25,26] in soil. In China, research on the determination of ammonia nitrogen is focusing on the optimization of previous methods [27,28,29]. For example, selecting the most appropriate type and concentration of extractant and improving extraction time [27,30]. However, few studies have proposed new methods to determine soil ammonia nitrogen. Furthermore, the absence of a unified national or industrial standard method has led to the use of multiple extraction methods. The traditional indophenol blue method uses 2 mol·L^−1^ KCl as the extractants, and previous studies have proved that 1 mol·L^−1^ KCl [27,29,31], 2 mol·L^−1^ KCl [32], and 2 mol·L^−1^ NaCl [27] can also be used as extractants.

A large number of rapid detection kits for ammonia nitrogen have emerged on the market. These kits are combined with spectrophotometry to determine ammonia nitrogen rapidly and quantitatively in water [33,34]. The soil ammonia nitrogen determination kits available on the market are not very effective because these kits are rarely compared with traditional methods [35,36]. Our lab has established a kit method for the rapid detection of ammonia nitrogen in water by improving salicylic acid spectrophotometry and optimizing the reaction temperature duration and pH conditions in 2015 [37], and the kit has been put into production by a company. This kit detection method is not significantly different from the national standard method. It enhances the anti-interference ability and effectively shortens the detection time. Further, we aimed to expand the application of this rapid detection kit to soil ammonia nitrogen determination because it is not significantly different from the national standard method.

Therefore, the objective of this study is to establish a rapid determination method for measuring soil ammonia nitrogen with a rapid detection kit [37] and microplate reader and to select suitable extractants which will improve the determination efficiency, reduce the usage of chemical reagents, and protect the health of the ecological environment. We believe our study will provide data to support the rapid determination technology for soil ammonia nitrogen.

## 2. Materials and Methods

### 2.1. Soil Samples

The present study was conducted in long-term fertilization paddy fields in the Fuyang (30°05′ N, 120°11′ E) and Yuhang districts (30°28′ N, 119°92′ E) of Hangzhou and the Jiande (29°28′ N, 119°16′ E) and Tonglu (29°79′ N, 119°68′ E) counties of Hangzhou, Zhejiang Province, in the subtropical region of Southern China. Common agricultural practices were applied at all study sites. The study sites had a history of five-year rice–wheat crop rotations. The fertilization treatments and fertilizer amounts of the soil samples are shown in Table 1. the soil samples were collected after rice harvesting by adopting a five-point sampling method. The soil samples collected from the topsoil (0–20 cm) were placed in an ice box and taken to the laboratory. Under laboratory conditions, the samples were mixed evenly and divided into two subsamples. One subsample was stored at −80 °C in a refrigerator for microbiological research, while the other subsample was air-dried and used to determine the physical and chemical properties of the soil after being ground and passed through a 2-mm sieve.

### 2.2. Main Instruments

The instruments used in the present study were a full wavelength microplate reader (Biotechnology instruments, Limited Company), a JM-B5003 electronic balance (0.001 g, Zhuji Chaoze Equipment Limited Company), and a Hz-9211kc constant temperature oscillator (Taicang science and education equipment factory).

### 2.3. Main Reagents

The reagents used in the present study were ammonia sulfate, sodium nitroferricyanide, sodium hydroxide, disodium hydrogen phosphate, sodium phosphate, sodium hypochlorite solution, potassium sodium tartrate, and ethylene diamine tetraacetic acid. All the reagents were purchased from Shanghai Aladdin Biochemical Technology Limited Company. A rapid detection kit (including liquid reagent and solid reagent in which the salicylic acid, sodium nitroferricyanide, NaDCC, borax, and sodium carbonate are mixed in a mass ratio of 0.5:1.0:0.05:2.5:25) was purchased from Zhejiang Luheng Environmental Technology Limited Company [37].

### 2.4. Reagent Configuration

Ammonia nitrogen standard storage solution (ρ = 100 mg·L^−1^): dissolve 0.4717 g dry ammonia sulfate in water and dilute to 1 L with water in a volumetric flask.

Ammonia nitrogen standard solution (ρ = 2 mg·L^−1^): add 2 mL ammonia nitrogen standard storage solution into a 100 mL volumetric flask and dilute to the mark.

Phenol solution: weigh 10 g phenol and 100 mg sodium nitroferricyanide and dissolve them in water to make a final volume of 1 L in a volumetric flask. This reagent is unstable and must be stored in a brown bottle at 4 °C.

Sodium hypochlorite alkaline solution: dissolve 10 g sodium hydroxide, 7.06 g disodium hydrogen phosphate, 31.8 g sodium phosphate, and 10 mL of 52.5 g·L^−1^ sodium hypochlorite in water to make a final volume of 1 L in a volumetric flask. This solution must also be stored in brown bottles at 4 °C.

Masking agent: mix 50 mL of 400 g·L^−1^ potassium sodium tartrate in 50 mL of 100 g·L^−1^ disodium EDTA solution in equal volume. Add 0.5 mL of 10 mol·L^−1^ sodium hydroxide solution to every 100 mL of mixed solution.

### 2.5. Kit Method

Weigh 5 g of air-dried soil and extract it with 25 mL of 1 mol·L^−1^ KCl, 2 mol·L^−1^ KCl, or 2 mol·L^−1^ NaCl solution. Shake at a constant temperature of 25 °C and a speed of 230 r·min^−1^ for 1 h [12]. Immediately filter the solution into a 50 mL centrifuge tube and determine the ammonia nitrogen with nitrogen rapid detection kit. The specific steps should be undertaken according to the manufacturers’ instructions: take 4 mL of the soil-leaching solution into a 15 mL centrifuge tube, add 6 mL of reagent I, and shake. Add one bag of reagent II and shake it to homogenize. After reaction for 20 min, use a microplate reader to measure absorbance at 650 nm.

### 2.6. Indophenol Blue Colorimetry

Weigh 5 g of air-dried soil and extract it with 25 mL of 2 mol·L^−1^ KCl solution. Shake it at a constant temperature of 25 °C and a speed of 230 r·min^−1^ for 1 h. Filter the solution into a 50 mL centrifuge tube. Suck 2–10 mL of soil-leaching solution into a 50 mL volumetric flask, add 10 mL of KCL solution, 5 mL of phenol solution, and 5 mL of sodium hypochlorite alkaline solution. Shake and leave the solution at room temperature for 1 h. Next, add 1 mL of masking agent to dissolve the possible precipitates and dilute the solution to 50 mL with double distilled water. Finally, use a microplate reader to measure absorbance at 650 nm [12].

### 2.7. Preparation of Standard Working Curve

Standard curve of rapid detection kit: add 0 mL, 0.8 mL, 1.6 mL, 2.4 mL, 3.2 mL, and 4 mL of ammonia nitrogen standard solution into 15 mL centrifuge tubes and supplement to 4mL with 1 mol·L^−1^ KCl, 2 mol·L^−1^ KCl, and 2 mol·L^−1^ NaCl respectively. Shake the solution as mentioned in Section 2.5.

Standard curve of indophenol blue colorimetry: add 0 mL, 2 mL, 4 mL, 6 mL, 8 mL, and 10 mL ammonia nitrogen standard solution into 50 mL volumetric flasks and add 10 mL of 2 mol·L^−1^ KCl solution, 5 mL of phenol solution, and 5 mL of sodium hypochlorite basic solution. Shake the solution as mentioned in Section 2.6.

### 2.8. Statistical Analysis

SPSS version 26 for Windows was used to analyze the *t*-test. All the graphs were produced by Origin 2021, while all the tables were produced by Excel 2016.

## 3. Results and Discussion

### 3.1. Selection of Reaction Conditions (Time and Temperature) for the Determination of Soil Ammonia Nitrogen Using the Kit Method

Our previous studies (2015) have revealed that the optimal chromogenic time and temperature of the kit for determining ammonia nitrogen in water were 10 min and 25–30 °C, respectively [37]. In this study, the rapid detection kit was used to determine soil ammonia nitrogen. First, the optimal chromogenic time and temperature of the kit for determining soil ammonia nitrogen were researched.

**Figure 1:**
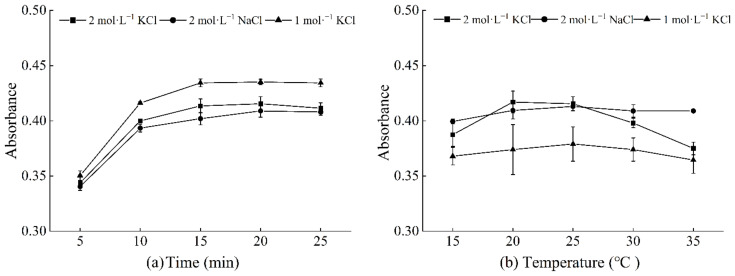
Change in absorbance with color development time (**a**) and temperature (**b**).

According to Section 2.5, the reagents were added and mixed thoroughly. After the reactions were carried out for 5 min, 10 min, 15 min, 20 min, or 25 min, the absorbance was measured at 650 nm using a microplate reader. The results showed that absorbance increased with the reaction time from 5 min to 20 min, but started to decrease gradually after 20 min (Figure 1a). Therefore, the optimal chromogenic time was selected as 20 min.

Following Section 2.5, the reagents were added and mixed well. After the reactions were carried out at 15 °C, 20 °C, 25 °C, 30 °C, or 35 °C for 20 min, the absorbance was measured at 650 nm using a microplate reader. The absorbance of the soil solution extracted using 2 mol·L^−1^ KCl started to stabilize at 20–25 °C. When the temperature was lower than 20 °C or higher than 20 °C, the absorbance began to decrease (Figure 1b). The absorbance of soil solutions extracted using 2 mol·L^−1^ NaCl and 1 mol·L^−1^ KCl reached their highest at 25 °C. Hence, the optimal chromogenic temperature was selected as 25 °C.

### 3.2. Comparison of Standard Working Curves of Soil Ammonia Nitrogen Reaction

The linear detection range of the indophenol blue colorimetry was 0–0.4 mg·L^−1^ for NH_4_^+^. The squared multiple correlation coefficient (R^2^) 0.9961 showed a highly significant correlation between ammonia nitrogen concentration and absorbance. When taking absorbance as abscissa and ammonia nitrogen concentration as ordinate, the standard working curve equation was y = 3.1411x − 0.2167. The linear detection range of the kit method was 0 mg·L^−1^ to 2 mg·L^−1^ for NH_4_^+^. When taking the absorbance value as abscissa and the ammonia nitrogen concentration as ordinate, the standard working curve equations of the 1 mol·L^−1^ KCl, 2 mol·L^−1^ KCl, and 2 mol·L^−1^ NaCl kit methods were y = 3.4354x − 0.6302, y = 2.7815x − 0.2654, and y = 2.6236x − 0.2219, respectively, and the R^2^ values were 0.9987, 0.9992 and 0.9996, respectively. The ammonia nitrogen concentration detection range of the kit method was larger than that of indophenol blue colorimetry. Additionally, the squared multiple correlation coefficient of the standard working curve was higher than that of the indophenol blue method, and the R^2^ values of standard working curves of 2 mol·L^−1^ KCl and 2 mol·L^−1^ NaCl were both 0.999, which indicates their higher accuracy.

### 3.3. Comparative Study on the Determination of Soil Ammonia Nitrogen Using the Kit Method and the Indophenol Blue Method

#### 3.3.1. Comparison of Determination Results of Ammonia Nitrogen in Soils with Different Nitrogen Contents

Soil samples from FY1–FY4 contained high amounts of ammonia nitrogen, while samples from FY5 and JD1–JD3 contained low amounts of ammonia nitrogen. The chemical properties of the soil samples are shown in Table 2. The FY1–FY4 and JD1–JD3 samples belonged to acid soils with organic matter ranging from 18.53 g·kg^−1^ ± 1.49 g·kg^−1^ to 32.31 g·kg^−1^ ± 0.55 g·kg^−1^ and available phosphorus ranging from 10.83 g·kg^−1^ ± 0.63 g·kg^−1^ to 28.33 g·kg^−1^ ± 0.48 g·kg^−1^. In addition, the available potassium of the FY1–FY5 samples was 32.28 mg·kg^−1^ ± 1.56 mg·kg^−1^ to 46.99 mg·kg^−1^ ± 2.56 mg·kg^−1^, while for samples JD1–JD3 it was 230.46 mg·kg^−1^ ± 12.17 mg·kg^−1^ to 256.20 mg·kg^−1^ ± 13.59 mg·kg^−1^. After extracting the soil samples with 2 mol·L^−1^ KCl, 2 mol·L^−1^ NaCl, and 1 mol·L^−1^ KCl, the soil ammonia nitrogen was determined using the kit method (Table 3), and the results were compared with the traditional indophenol blue colorimetry method. Tests for each sample were performed in triplicate.

According to the *t*-test results (Table 3), four samples (FY1, FY3, FY5, and JD3) showed significant differences between ammonia nitrogen determined using the 2 mol·L^−1^ KCl extraction kit method and indophenol blue colorimetry (*p* < 0.05). Sample FY1 and sample FY3 contained high amounts of ammonia nitrogen, and sample FY5 and sample JD3 contained low amounts of ammonia nitrogen, indicating that 2 mol·L^−1^ KCl was not suitable for determining whether soil contain s high or low amounts of ammonia nitrogen. A possible reason is that 2 mol·L^−1^ KCl as an extractant increases the total ionic strength of the determination solution and other impurities, which react with the masking agent in the kit and affect the determination [38,39]. Five samples (FY1–FY5) showed significant differences between ammonia nitrogen determination using the 2 mol·L^−1^ NaCl extraction kit method and indophenol blue colorimetry (*p* < 0.05). The differences in the results were mainly due to high concentrations of ammonia nitrogen. Gong et al. (2006) extracted soil with 20% NaCl solution to determine ammonia nitrogen [13]. Therefore, we believe that 2 mol·L^−1^ NaCl may not completely extract ammonia nitrogen from the soil, leading to inconsistent results. Moreover, most of the researchers used KCl solution as an extractant rather than NaCl solution [28,29]. Similarly, our results indicated that KCl is more suitable for soil extraction.

Alternatively, no significant difference between the results of the 1 mol·L^−1^ KCl extraction kit method and indophenol blue colorimetry were observed (*p* > 0.05), indicating the feasibility of using the 1 mol·L^−1^ KCl extraction kit method for ammonia nitrogen determination in soil with high and low ammonia nitrogen contents.

#### 3.3.2. Comparison of Determination Results of Ammonia Nitrogen in Soils with Different Chemical Properties

The chemical properties of the soil samples with different pH values, organic matter content, available phosphorus content, and available potassium content are shown in Table 4. The samples had acidic and neutral soil pH values with organic matter content ranging from 16.21 g·kg^−1^ ± 0.81 g·kg^−1^ to 42.97 g·kg^−1^ ± 2.15 g·kg^−1^ and available phosphorus content ranging from 2.60 g·kg^−1^ ± 0.13 g·kg^−1^ to 35.66 g·kg^−1^ ± 1.78 g·kg^−1^. In addition, the available potassium content of the JD4–JD6 and TL1–TL3 samples ranged from 201.10 mg·kg^−1^ to 285.87 mg·kg^−1^, while the available potassium content of samples FY6–FY8 and YH1–YH3 ranged from 29.00 mg·kg^−1^ ± 1.45 mg·kg^−1^ to 65.37 mg·kg^−1^ ± 3.26 mg·kg^−1^.

After extracting soil samples with 2 mol·L^−1^ KCl, 1 mol·L^−1^ KCl, and 2 mol·L^−1^ NaCl, ammonia nitrogen was determined using the kit method (Table 5), and the results were compared with those obtained using classical indophenol blue colorimetry. According to the results of the *t*-tests, no significant difference was observed between the 1 mol·L^−1^ KCl extraction kit method and indophenol blue colorimetry (*p* > 0.05). However, three samples (JD4, FY6, and YH3) showed significant differences between their ammonia nitrogen as determined by the 2 mol·L^−1^ KCl extraction kit method and by indophenol blue colorimetry (*p* < 0.05). Samples JD4, FY6, and YH3 were collected from Jiande county, Fuyang district and Yuhang district, respectively, and the chemical properties of these three soils are significantly different, indicating that the 2 mol·L^−1^ KCl extraction kit method is not suitable for determining the paddy soil ammonia nitrogen.

In addition, four samples (TL1, FY6, YH1, and YH3) showed significant differences in ammonia nitrogen as determined using the 2 mol·L^−1^ NaCl extraction kit method and indophenol blue colorimetry (*p* < 0.05). The chemical properties of these four soils are different, which indicates that the 2 mol·L^−1^ NaCl extraction kit method is not suitable for determining the paddy soil ammonia nitrogen.

The above results indicated that the 1 mol·L^−1^ KCl extraction kit method could be substituted for indophenol blue colorimetry and applied to determine the ammonia nitrogen content in various types of paddy soil. A previous study showed that when the concentration of the KCl solution reached 1 mol·L^−1^, the ammonia nitrogen in the soil sample should be fully extracted [27]. However, another study revealed that 1 mol·L^−1^ KCl is the most suitable extractant [31], while a further study revealed that 2 mol·L^−1^ KCl is most suitable extractant [32]. In the present study, 1 mol·L^−1^ KCl was determined to be the most suitable extractant; 2 mol·L^−1^ KCl and 2 mol·L^−1^ NaCl may contain more interference substances due to their high concentration [39], and these react with the masking agent in the kit to affect the results. However, our study discussed the feasibility of the kit method for determining the paddy soil ammonia nitrogen.

### 3.4. Precision Comparison between Kit Method and Indophenol Blue Method

Twelve samples were analyzed using the 1 mol·L^−1^ KCl extraction kit method and indophenol blue colorimetry, and each sample contained five replicates. The precision of indophenol blue colorimetry and of the kit method is shown in Table 6. The relative standard deviations of indophenol blue colorimetry and the 1 mol·L^−1^ KCl extraction kit method were between 4.6% and 31.4% and between 0.3% and 15.80%, respectively. Additionally, the average relative deviations of indophenol blue colorimetry and the 1 mol·L^−1^ KCl extraction kit method were 15.2% and 7.0%, respectively. A large relative standard deviation indicates a lower degree of precision. The relative standard deviation of the kit method was significantly smaller than that of the indophenol blue method, which means that the precision of the kit method is higher than that of the indophenol blue colorimetry method. High soil heterogeneity may lead to a high relative standard deviation.

### 3.5. Recovery Rate and Detection Limit of Kit Method

The ammonia nitrogen content of the different soil samples collected from Jiande county, Tonglu county, and Yuhang district was determined. The ammonia nitrogen standard solutions with a concentration of 1 mol·L^−1^ KCl as solvent were added to the soil samples, and the volume of extractant added was reduced accordingly. The standard adding recovery rate of the kit method ranged from 89.30% to 118.23%. Compared with other ammonia nitrogen determination methods [19], the kit method has a high standard addition recovery rate, which indicates that the kit method has high accuracy (Table 7).

The standard deviation was calculated according to the ammonia nitrogen results of twelve blank samples, and three times blank standard deviation was used as the detection limit estimation value. The standard deviation of ammonia nitrogen in blank was determined to be 2.47% using the 1 mol·L^−1^ KCl kit–microplate reader method, and the detection limit was 0.074 mg·L^−1^ (lower than the World Health Organization standard value of 10 mg/L) [40], which means the dispersion was low and the data were stable.

## 4. Conclusions

In present study, 1 mol·L^−1^ KCl, 2 mol·L^−1^ KCl, and 2 mol·L^−1^ NaCl solutions were used to extract soil ammonia nitrogen. The method of determining soil ammonia nitrogen using a rapid detection kit was optimized by optimizing the time to 20 min and the temperature to 25 °C. The results showed that no significant difference was found between the ammonia nitrogen measured using the 1 mol·L^−1^ KCl kit method and the ammonia nitrogen measured using indophenol blue colorimetry. In addition, the 1 mol·L^−1^ KCl kit method has several advantages over the traditional method due to its linearity (kit method: 0.9987, indophenol blue colorimetry: 0.9961), precision (kit method: 0.3–15.80%, indophenol blue colorimetry: 4.6–31.4%), recovery (kit method: 89.30–118.23%), and accuracy. In addition, the kit method can be applied to determine soil ammonia nitrogen and various physical and chemical properties. Furthermore, 96 samples can be determined simultaneously by combining this method with full wavelength microplate reader colorimetry, which dramatically shortens the analysis time, reduces the number of operation steps, and enables the rapid mass determination of samples. The 1 mol·L^−1^ KCl kit method is also applicable for paddy soil. Further studies are needed to explore the use of the kit method with other types of soil.

## Figures and Tables

**Table 1:** The fertilization treatment and fertilizer amount of soil samples from Fuyang and Yuhang districts, and from Jiande and Tonglu counties.

Sample	Treatment	Fertilizer Amount (kg·hm^−2^)	Sample	Treatment	Fertilizer Amount (kg·hm^−2^)
FY1	Formula fertilizer	456	JD3	Womeike soil conditioner	9000
FY2	Organic fertilizer	288	JD4	Mulanqing soil conditioner	9000
FY3	Wanli Shennong slow-release fertilizer	373.5	JD5	Mulanqing soil conditioner	4500
FY4	Xinlianxin slow-release fertilizer	373.5	JD6	Tebeigai soil conditioner	750
FY5	Yantai Longdeng slow-release fertilizer	373.5	TL1	Mulanqing soil conditioner	9000
FY6	Jindaaohe slow-release fertilizer	373.5	TL2	Tebeigai soil conditioner	750
FY7	Yangfeng baomo slow-release fertilizer	373.5	TL3	Tebeigai soil conditioner	1500
FY8	CK	0	YH1	Organic slow-release fertilizer	276
JD1	CK	0	YH2	Conventional fertilization	314
JD2	Tebeigai soil conditioner	1500	YH3	Yongxiao slow-release fertilizer	276

Note: JD1–JD6 are soil samples from Jiande county; TL1–TL3 are soil samples from Tonglu county; FY1–FY8 are soil samples from Fuyang district; YH1–YH3 are soil samples from Yuhang district.

**Table 2:** Chemical properties of soil samples collected from Fuyang and Jiande districts.

Sample	pH	OM (g·kg^−1^)	AP (mg·kg^−1^)	AK (mg·kg^−1^)
FY1	5.46 ± 0.08	22.64 ± 0.34	16.22 ± 0.24	32.28 ± 1.56
FY2	5.28 ± 0.15	28.65 ± 0.43	28.33 ± 0.48	36.12 ± 1.68
FY3	5.10 ± 0.23	30.91 ± 1.46	26.00 ± 0.54	34.06 ± 1.33
FY4	5.22 ± 0.15	30.85 ± 0.58	11.30 ± 0.32	32.28 ± 1.34
FY5	5.13 ± 0.52	32.51 ± 1.25	16.24 ± 0.15	46.99 ± 2.56
JD1	4.26 ± 0.06	21.86 ± 0.39	12.27 ± 0.17	230.46 ± 12.17
JD2	5.19 ± 0.49	32.31 ± 0.55	11.97 ± 0.22	256.20 ± 13.59
JD3	5.90 ± 0.33	18.53 ± 1.49	10.83 ± 0.63	250.40 ± 10.35

Note: FY1–FY5 are soil samples from Fuyang district; JD1–JD3 are soil samples from Jiande district. OM: organic matter; AP: available phosphorus; AK: available potassium.

**Table 3:** Comparison of soil ammonia nitrogen determination using kit method and indophenol blue colorimetry (*n* = 3).

Sample	Ammonia Nitrogen (mg·kg^−1^)				*t*-Test Results		
	T1	T2	T3	T4	*p*1	*p*2	*p*3
FY1	67.72 ± 0.80	46.59 ± 0.62	49.73 ± 2.65	68.47 ± 0.27	0.00	0.00	0.20
FY2	72.45 ± 6.69	65.29 ± 2.10	46.83 ± 1.25	71.06 ± 1.42	0.15	0.00	0.74
FY3	58.81 ± 6.15	41.40 ± 1.55	44.18 ± 2.66	56.53 ± 0.86	0.03	0.02	0.59
FY4	44.29 ± 6.35	36.48 ± 1.09	32.41 ± 2.44	45.57 ± 0.40	0.10	0.04	0.75
FY5	4.68 ± 0.45	3.65 ± 0.31	5.92 ± 0.09	5.80 ± 0.60	0.03	0.04	0.06
JD1	6.35 ± 0.10	13.83 ± 0.55	10.88 ± 1.37	9.45 ± 0.77	0.081	0.632	0.285
JD2	4.68 ± 0.76	3.53 ± 0.27	4.90 ± 0.57	5.19 ± 0.16	0.07	0.70	0.37
JD3	9.56 ± 0.44	13.83 ± 0.55	10.88 ± 1.37	9.45 ± 0.77	0.00	0.19	0.83

Note: FY1–FY5 are soil samples collected from Fuyang district; JD1–JD3 are soil samples from Jiande district. T1: indophenol blue colorimetry; T2: 2 mol·L^−1^ KCl extraction kit method; T3: 2 mol·L^−1^ NaCl extraction kit method; T4: 1 mol·L^−1^ KCl extraction kit method; *p1*: *t*-test results for indophenol blue colorimetry and 2 mol·L^−1^ KCl extraction kit; *p2*: *t*-test results for indophenol blue colorimetry and 2 mol·L^−1^ NaCl Extraction Kit; *p3*: *t*-test results for indophenol blue colorimetry and 1 mol·L^−1^ KCl extraction kit.

**Table 4:** Chemical properties of soil samples from Fuyang and Yuhang districts, and from Jiande and Tonglu counties.

Sample	pH	OM (g·kg^−1^)	AP (mg·kg^−1^)	AK (mg·kg^−1^)
JD4	4.48 ± 0.22	16.66 ± 0.83	11.81 ± 0.59	285.87 ± 14.29
JD5	4.06 ± 0.20	16.44 ± 0.82	12.46 ± 0.62	256.01 ± 12.80
JD6	4.58 ± 0.23	16.21 ± 0.81	15.39 ± 0.77	201.10 ± 10.06
TL1	7.50 ± 0.38	22.08 ± 1.10	15.15 ± 0.76	221.36 ± 11.07
TL2	7.37 ± 0.37	31.47 ± 1.57	14.22 ± 0.71	243.30 ± 12.17
TL3	7.17 ± 0.36	29.41 ± 1.47	14.60 ± 0.73	278.80 ± 13.94
FY6	5.68 ± 0.29	20.80 ± 1.04	2.60 ± 0.13	29.00 ± 1.45
FY7	5.22 ± 0.26	30.20 ± 1.51	26.90 ± 1.35	41.20 ± 2.06
FY8	5.09 ± 0.25	31.66 ± 1.58	35.66 ± 1.78	43.06 ± 2.15
YH1	5.24 ± 0.26	37.30 ± 1.87	9.90 ± 0.20	57.32 ± 2.87
YH2	5.17 ± 0.26	37.76 ± 1.89	10.00 ± 0.50	65.37 ± 3.26
YH3	5.69 ± 0.29	42.97 ± 2.15	20.64 ± 0.41	62.85 ± 3.14

Note: JD4–JD6 are soil samples from Jiande county; TL1–TL3 are soil samples from Tonglu county; FY6–FY8 are soil samples from Fuyang district; YH1–YH3 are soil samples from Yuhang district. OM: organic matter; AP: available phosphorus; AK: available potassium.

**Table 5:** Comparison between determination results of Kit Method and Indophenol Blue Colorimetry (*n* = 3).

Sample	Ammonia Nitrogen (mg·kg^−1^)				*t*-Test Results		
	T1	T2	T3	T4	*p*1	*p*2	*p*3
JD4	9.55 ± 1.73	5.57 ± 0.36	9.65 ± 0.41	9.48 ± 0.59	0.00	0.86	0.92
JD5	37.80 ± 9.15	25.86 ± 1.80	26.64 ± 1.87	30.09 ± 1.09	0.09	0.11	0.22
JD6	5.72 ± 0.80	4.28 ± 0.86	6.46 ± 0.37	5.42 ± 0.34	0.11	0.07	0.05
TL1	6.18 ± 1.94	6.32 ± 1.06	4.60 ± 0.20	5.62 ± 0.71	0.86	0.02	0.37
TL2	6.45 ± 0.55	6.35 ± 0.23	7.91 ± 1.39	6..10 ± 0.24	0.79	0.17	0.40
TL3	6.64 ± 0.36	6.11 ± 0.32	6.90 ± 1.92	6.00 ± 0.29	0.13	0.83	0.07
FY6	35.95 ± 0.51	31.79 ± 0.51	31.95 ± 1.09	37.05 ± 0.12	0.14	0.16	0.67
FY7	24.60 ± 4.77	24.43 ± 0.68	21.96 ± 2.88	24.12 ± 1.42	0.96	0.46	0.88
FY8	56.73 ± 5.45	42.92 ± 2.66	41.78 ± 2.21	54.65 ± 3.39	0.02	0.01	0.61
YH1	14.88 ± 0.68	11.92 ± 0.46	10.78 ± 0.05	14.67 ± 0.04	0.06	0.02	0.87
YH2	11.98 ± 0.27	10.61 ± 1.21	11.11 ± 0.05	9.83 ± 2.32	0.13	0.10	0.19
YH3	4.46 ± 0.95	10.04 ± 0.10	6.91 ± 0.18	4.17 ± 0.26	0.01	0.04	0.64

Note: JD4–JD6 are soil samples from Jiande county; TL1–TL3 are soil samples from Tonglu county; FY6–FY8 are soil samples from Fuyang district; YH1–YH3 are soil samples from Yuhang district. T1: indophenol blue colorimetry; T2: 2 mol·L^−1^ KCl extraction kit method; T3: 2 mol·L^−1^ NaCl extraction kit method; T4: 1 mol·L^−1^ KCl extraction kit method; *p*1: *t*-test results for indophenol blue colorimetry and 2 mol·L^−1^ KCl extraction kit; *p*2: *t*-test results for indophenol blue colorimetry and 2 mol·L^−1^ NaCl extraction kit; *p*3: *t*-test results for indophenol blue colorimetry and 1 mol·L^−1^ KCl extraction kit.

**Table 6:** Precision of indophenol blue colorimetry and kit method (*n* = 5).

Sample	Relative Standard Deviation (%)	
	T1	T4
**JD4**	18.10	6.20
**JD5**	13.90	6.20
**JD6**	23.80	10.40
**TL1**	31.40	12.60
**TL2**	8.60	3.90
**TL3**	18.50	4.80
**FY6**	9.60	6.20
**FY7**	19.40	5.90
**FY8**	10.70	3.50
**YH1**	4.60	0.30
**YH2**	2.30	15.80
**YH3**	21.40	8.00
**Average**	15.20	7.00

Note: JD4–JD6 are soil samples from Jiande county; TL1–TL3 are soil samples from Tonglu county; FY6–FY8 are soil samples from Fuyang district; YH1–YH3 are soil samples from Yuhang district. T1: indophenol blue colorimetry; T4: 1 mol·L^−1^ KCl extraction kit method.

**Table 7:** Standard addition recovery test using the kit method.

Sample	Standard Addition Concentration (mg·L^−1^)	Measured Value (mg·L^−1^)			Standard Adding Recovery Rate (%)
		1	2	3	
JD3	1.00	0.91	0.82	0.95	89.30 ± 6.71
TL2	1.00	1.19	1.16	1.19	118.23 ± 1.52
YH2	1.00	1.05	0.94	1.09	102.35 ± 7.84

Note: JD3 is soil from Jiande county; TL2 is soil from Tonglu county; YH2 is soil from Yuhang district.

## Data Availability

Not applicable.
